# Timing Determination of Invasive Fungal Infection Prophylaxis According to Immune Function in HSCT Patients

**DOI:** 10.3389/fmicb.2018.00370

**Published:** 2018-03-02

**Authors:** Jiexian Ma, Yingwei Hu, Min Wu, Xiaoqin Wang, Yanhui Xie

**Affiliations:** ^1^Department of Hematology, Huashan Hospital Affiliated to Fudan University, Shanghai, China; ^2^Department of Hematology, Huadong Hospital Affiliated to Fudan University, Shanghai, China; ^3^Shanghai Key Laboratory of Clinical Geriatric Medicine, Shanghai, China

**Keywords:** IgG, NK cell count, invasive fungal infection, HSCT, ROC curve

## Abstract

Patients who receive a hematopoietic stem cell transplantation (HSCT) exhibit an immune defect after recovering from neutropenia. The current guidelines do not recommend fungal prophylaxis in these patients, except for grades III to IV GVHD in HSCT. Thus, the timing for the initiation and cessation of IFI prophylaxis in immune-compromised patients remains a challenging endeavor. We retrospectively analyzed patients who received auto or allo-HSCT and monitored their immune function after recovering from neutropenia by measuring the levels of IgG, IgA, IgM, as well as the number of T, B, NK cells. We found that the level of IgG and NK cell count exhibited a significant difference with the incidence of IFI by logistic regression (*p* = 0.000 vs. 0.000, respectively) and conditional logistic regression (*p* = 0.009 vs. *p* = 0.002). The initiation of IFI prophylaxis was determined to be IgG < 7 mg/mL and NK cell count < 6.5 × 104/mL by an receiver operating characteristic curve separately. Tests in parallel increased the test sensitivity and specificity. Thus, the optimal timing for initiating prophylaxis in patients after HSCT could be IgG < 7 mg/mL or NK cell count < 6.5 × 104/mL. Future large-scale prospective clinical trials are required to verify these findings. Patients who are immuno-compromised after auto or allo-HSCT may benefit from a lower fungi infection incidence with immune surveillance and proper fungal prophylaxis.

## Introduction

Invasive fungal infection (IFI) is an important cause of morbidity and mortality among patients with hematological malignancies, particularly in those who receive intensive cytotoxic chemotherapy, immunosuppressive drugs, or hematopoietic stem cell transplantation (HSCT) (Lin et al., 2001; Gavalda et al., 2005; Oliveira-Coelho et al., 2015). The prevalence of invasive fungal infection has steadily increased over the past few decades, mainly due to the advent of HSCT, as well as the increased use of chemotherapy and immunosuppressive drugs for hematological patients (Kontoyiannis et al., 2010; Pagano et al., 2010). Invasive aspergillosis (IA) is the most common fungal infection, and a leading cause of mortality in this patient population (Kontoyiannis et al., 2010; Pagano et al., 2010). Moreover, mucormycosis is the second most common IFI in patients with hematological malignancies (Kontoyiannis et al., 2010; Pagano et al., 2010). The Analyses of The Transplant-Associated Infection Surveillance Network (TRANSNET) database showed that the cumulative annual incidence of IFIs was 7.7, 8.1, 5.8, and 1.7 in every 100 transplants for matched-unrelated allogeneic, mismatch-related allogeneic, matched-related allogeneic, and autologous HSCT patients, respectively (Gavalda et al., 2005). In addition, a study from Japan showed that the incidence of IFI was 5.4% in allogeneic HSCT patients, 0.4% in autologous HSCT patients, and 0.8% in patients receiving chemotherapy alone (Neofytos et al., 2009). Indeed, IFIs were very difficult to eradicate in HSCT patients. The reported mortality rate of IFI ranges from 50% in patients with leukemia or lymphoma to 87% in HSCT patients (Kurosawa et al., 2012). In the TRANSNET database, the estimated post-HSCT 1-year survival for invasive candidiasis and invasive aspergillosis is only 33 and 25%, respectively (Kontoyiannis et al., 2010).

Due to the high mortality associated with IFI, prophylactic treatment of IFIs is extremely important. Recent studies have demonstrated that the routine prophylactic use of fluconazole after HSCT decreased the risk of invasive candidiasis to a low incidence of 1.1–5% (Lin et al., 2001; Martino et al., 2002; Jantunen et al., 2004; Kriengkauykiat et al., 2011; Mellinghoff et al., 2018). Since unnecessary prophylaxis can induce drug resistance and cause side effects (Martín-Peña et al., 2014), it is extremely important to identify the risk factors for IFI. The identified risk factors of IFI include age, sex, immunosuppression, venous catheter, diabetes, neutropenia, mucositis, steroid use, graft-versus-host disease, and cytomegalovirus infection (Lehrnbecher et al., 2013; Camargo et al., 2015). In immunocompromised patients (e.g., those who received auto or allo-HSCT) the timing for the initiation and cessation of IFI prophylaxis remains a challenging endeavor. Most clinicians rely on their experience since the guidelines only recommend that patients who suffer from grades III to IV GVHD should receive anti-fungal prophylaxis following an allo-HSCT (Chinese Invasive Fungal Infection Working Group, 2017). Moreover, many patients who do not require prophylaxis experience severe IFI after HSCT. We retrospectively collected 141 hematological patients who received auto- or allo-hematological transplantation and did not require IFI prophylaxis following recovery from neutropenia according to the guidelines. During the follow up, we found that 25 patients had IFI, and the incidence of IFI was high, at 17.7%.

The establishment of an index and relationship with IFI in patients after HSCT appears necessary. We retrospectively analyzed the data of 141 patients after allo- or auto-HSCT regarding their immune function based on immunoglobulin levels and CD4+, CD8+, NK cell counts, then analyzed the incidence of IFI in these patients. Our study attempted to explore the relationship between immune function and IFI by a logistic regression analysis, and we also analyzed the possible cut-off point for initiating fungal prophylaxis to formulate an appropriate IFI prophylaxis strategy.

## Materials and Methods

### Study Patient Population

We retrospectively reviewed the medical records of 141 patients (aged ≥ 16 years) who received autogeneic (130 patients) or allogeneic HSCT (11 patients) between January 1^st^, 2012 and December 31^st^, 2016 in Huadong and Huashan Hospital, Shanghai, China. The recorded clinical data included age, gender, disease diagnosis, type of transplant, conditioning regimen, GVHD, IFI and other clinical complications, which was all retrospectively collected. The retrospective review of medical records was approved by the Institutional Ethical Committee of Huadong and Huashan Hospital (Ethical Committee Number: 2017K071; Approved: October, 2017) in agreement with the Helsinki Declaration of 1975, revised in 2008. All patients in this study provided informed consent in accordance with the Declaration of Helsinki.

### Transplant Details and Conditioning Regimens

We established regimens in accordance with the patient’s disease type and sensitivity to the drugs. The regimens principally consisted of standard chemotherapy combined with novel drugs (e.g., rituximab, bortezomib, fludarabine, or cladribine) to maximally reduce the tumor burden. Subsequently, autologous HSCT was used as consolidation therapy. Autologous HSCT contains stem cell mobilization, conditioning regimens, stem cell re-transfusion, bone marrow arrest and recovery. The conditioning regimen for autologous transplantation consisted of 60 mg/(m^2^.d) d-2 mitoxantrone, 60 mg/(kg.d) d-2 CTX and 30 mg/(kg.d) d-2 VP-16 for lymphoma, 60 mg/(m^2^.d) d-2 mitoxantrone, 60 mg/(kg.d) d-2 CTX, and 5 g/(m^2^.d) d-2 Ara-C for leukemia, and 200 mg/(m^2^.d) d-2 Mel for myeloma. We often used rituximab (375 mg/m^2^) once before stem cell collection to further irradiate the tumor cells in patients with lymphoma. If the patient suffered from refractory leukemia, we administered fludarabine (25 mg/m^2^) or cladribine (5 mg/m^2^) for 5 days. The allo-HSCT often contained ATG and high-dose CTX to reach sufficient levels of immune-suppression. The regimen for allo-HSCT often consisted of 30 mg/(m^2^.d) × 2d fludarabine, 60 mg/(kg.d) × 2d CTX and 200/(m^2^.d) × 1d MeCCNu, as well as 2.5 mg/(kg.d) × 4d ATG.

### Immune Function Monitoring

According to the guidelines (De Pauw et al., 2008; Fleming et al., 2014), after recovering from neutropenia, only patients who suffered from III to IV graft-versus-host diseases in allo-HSCT should receive antifungal prophylaxis. None of the patients who entered into this study required antifungal prophylaxis after neutropenia recovery according to the guidelines. Following transplantation, the patients must receive humoral and cellular immunity functional surveillance every 3 months. The assessment of humoral immune function involved measuring the level of IgG, IgM, IgA levels using a turbidimetric immunoassay. Cellular immune function was assessed according to the CD4+/CD8+, CD4+, CD8+, CD19+, and NK cell count proportions measured by flow cytometry (BD corporation). A total of 50,000 cells were counted each time. The cell counts were calculated using a peripheral blood test and the cell count proportion was measured by flow cytometry.

### Clinical Evaluation and Definition of IFI

According to the revised definitions of the European Organization for Research and Treatment of Cancer (EORTC)/the National Institute of Allergy and Infectious Diseases Mycoses Study Group (MSG) Consensus Group (Fleming et al., 2014), a probable IFI requires the presence of both a clinical and mycological criterion. Proof of an IFI is demonstrated by the observation of fungal elements by microscopic examination or positive results from a fungal culture of diseased tissue or blood. In our study, patients with clinical criteria of IFI (e.g., typical signs on lung computed tomography, images showing sinonasal, or CNS infection) required supplementary evidence from mycological criteria (e.g., positive results from cytology, direct microscopic examinations, fungal culture or an indirect test for detecting GM antigen in plasma, serum, bronchoalveolar lavage fluid, or cerebrospinal fluid). Most of the probable IFI patients recovered after anti-fungal therapy, and only two patients died of infection.

### Follow-Up of Patients

For the follow-up of auto-HSCT or allo-HSCT, the patients received a total body examination, including the collection of clinical data (e.g., name, sex, age, history as well as chronic disease, blood test, total body CT scan, bone marrow biopsy, and immune function measurements) every 3 months during the first year and every 6 months in the second year. Data regarding immune function was collected every 3 months during the first year after HSCT, and the data was recorded as the average data for all assessments of immune function.

### Statistical Analysis

SPSS version 20.0 (SPSS, Inc., Chicago, IL, United States) was used. Clinical characteristics, e.g., age, sex, disease type, neutrophil count, presence of a venous catheter, diabetes, a prior IFI history, transplant type, as well as whether the refractory diseases were analyzed by a *t*-test or Chi-square test. Possible predictors for IFI were retrospectively analyzed using a Chi-square test, and factors with *p* < 0.1 were analyzed with logistic regression models. The results are expressed as the odds ratios (OR) and their corresponding 95% confidence intervals (CIs). To increase the level of statistic efficiency, the patients’ age, whether they suffered from diabetes, and a prior history of IFI were judged as matching factors, then paired at a ratio of one case to three controls. A conditional logistic regression was also used to analyze the probable risk factors of an IFI. To determine the timing of effective anti-fungal prophylaxis after the calculation of risk factors for IFI, an receiver operating characteristic (ROC) curve was used to determine the cut-off point for IFI. Factors statistically significant (*p* < 0.05) in the univariate analysis were included in the multivariate analysis. Sensitivity, specificity, as well as positive and negative predictive values (PPV and NPV) were calculated for the threshold of risk factors. To increase the sensitivity and specificity of the test, testing in series and parallel were compared against the incidence of IFI.

## Results

### Clinical Characteristics of the Study Patients

In total, 141 patients who received auto-HSCT and allo-HSCT between January 1^st^, 2012 and December 31^st^, 2016 were analyzed. We collected the clinical characteristics and history of the patients, and all the risk factors for IFI, except immune situations, were comparable (*p* > 0.05) between the fungal and non-fungal infection groups. The clinical characteristics of the patients are summarized in **Table 1**.

**Table 1:** Clinical characteristic of patients who receive stem cell transplantation.

	Invasive fungal infection (25 patients)	Non-invasive fungal infection (116 patients)	*p*-value
Age (year)	51.00 (37.00–60.00)	53.00 (49.00–60.00)	0.161
Sex (F/M)	6/19	67/49	0.106
Disease type (lymphoma/leukemia/myeloma)	21/1/3	74/38/4	0.118
Neutrophil count (×10^9^/L)	4.0 (3.7–4.2)	4.6 (4.0–5.53)	0.078
Venous catheter (yes/no)	5/20	29/87	0.599
Diabetes (yes/no)	11/14	49/67	0.872
Prior history of IFI (yes/no)	3/22	10/106	0.596
Transplant type (auto-HSCT/allo-HSCT)	23/2	107/9	0.967
Refractory diseases (yes/no)	13/12	43/73	0.166

### Incidence of IFI

The overall cumulative IFI incidence was 17.73% (25/141), including 17 proven and eight probable cases before the patients received antifungal treatment. A total of two patients died of infection, and 23 patients recovered after receiving antifungal treatment. There were 130 patients who received auto-HSCT, and 11 patients who received allo-HSCT. The cumulative IFI incidence of auto-HSCT was 17.69% (23/130). The cumulative IFI incidence in allo-HSCT was 18.18% (2/11). The incidence of IFI post-auto-HSCT was higher than that reported in other clinical trials, the history of prior IFI (9.21%) in these patients was more common than that reported in other clinical trials (Gavalda et al., 2005). Since many patients (39.71%) were refractory to routine chemotherapy, additional drugs such as rituximab, bortezomib, fludarabine, and cladribine were administered to reduce the incidence of relapse. However, such drugs were found to damage the patients’ immune function and prolong immune reconstitution. Immune dysfunction in these patients was extremely common due to the widespread use of immunosuppressive drugs.

### Post-transplant Risk Factors for the Occurrence of IFI

We then analyzed the possible factors, including the levels of IgA, IgG, IgM, as well as CD4/CD8 T cell, NK cell, T cell, and B cell counts, to clarify whether these factors were correlated with a fungal infection. According to the range of the normal value, we also established variables, such as IgG < 7 mg/mL, IgA < 0.7 mg/mL, IgM < 0.4 mg/mL, and an absolute NK cell count < 7 × 10^4^/mL. We found that IgG < 7 mg/mL, IgA < 0.7 mg/mL, and the absolute NK cell count < 7 × 10^4^/mL was related to the IFI by a Chi-square test. We then used a logistic regression analysis and found that IgG < 7 mg/mL and the absolute NK cell count < 7 × 10^4^/mL exhibited a significant difference between the IFI and non-IFI group (*p* = 0.001 vs. 0.000, respectively). The ORs were 15.87 (3.93–62.5) for IgG < 7 mg/mL and 26.31 (6.67–111.11) for the NK cell count < 7 × 10^4^/mL, respectively (**Table 2**).

**Table 2:** Predictive factors for IFI by logistic regression.

Variables	*B*	OR	95% CI for OR	*p*
IgG < 7 mg/mL	-2.757	15.87	3.93–62.5	0.000
NK cell count < 7 × 10^4^/mL	-3.278	26.31	6.67–111.11	0.000
IgA < 0.7 mg/mL	-0.953	2.59	0.69–9.71	0.157

### Conditional Logistic Regression Analysis

Due to the wide range of 95%CI for the OR and confounding factors in this study, we used a conditional logistic regression with a paired test to increase the statistic efficiency. A case was defined as having an IFI, and a control was defined as not having IFI. Three were 25 IFI patients, and each IFI patient was matched with three non-IFI patients. The matched factors were judged to potentially confound the association between an IFI and possible risk factors. They included: (1) patients’ age; (2) whether they suffered from diabetes; (3) whether they have a prior history of IFI and controllable IFI before stem cell transplantation. Among the matched factors, only age was a continuous variable, a difference (caliper) of no more than 10 years for age in a pair. We then used a logistic regression analysis and also found that IgG < 7 mg/mL and absolute NK cell count < 7 × 10^4^/mL exhibited a significant difference between the IFI and non-IFI groups (*p* = 0.009 vs. 0.002, respectively), ORs were 10.53 (1.80–62.5) for IgG < 7 mg/mL and 27.03 (3.22–250) for the NK cell count < 7 × 10^4^/mL, respectively (**Table 3**).

**Table 3:** Predictive factors for IFI by conditional logistic regression.

Variables	*B*	OR	95% CI for OR	*p*
IgG < 7 mg/mL	-2.353	10.53	1.80–62.5	0.009
NK cell count < 7 × 10^4^/mL	-3.309	27.03	3.22–250	0.002
IgA < 0.7 mg/mL	-0.331	1.39	0.30–6.37	0.67

### ROC Curve to Calculate the Cut-Off Value for the Timing of Antifungal Infection Prophylaxis

**FIGURE 1:**
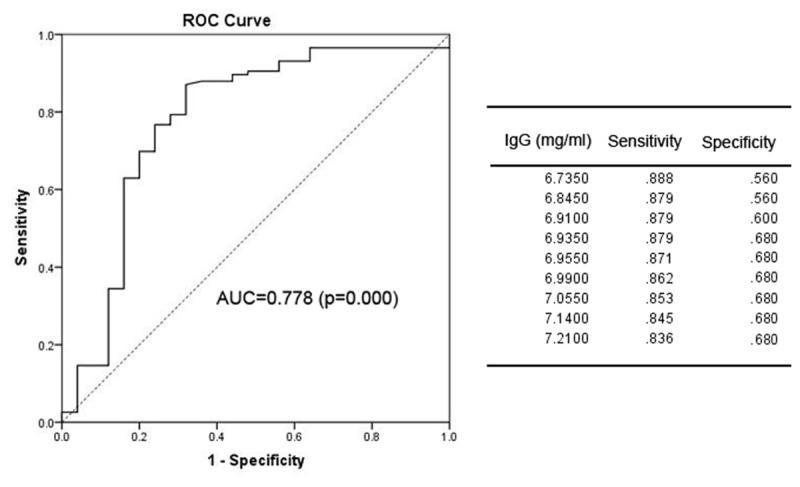
The receiver operating characteristic (ROC) analysis of IgG levels. The ROC curve and statistics table for the cut-off value of IgG levels in the patients’ peripheral blood for the group of patients with proven and probable invasive infection.

**FIGURE 2:**
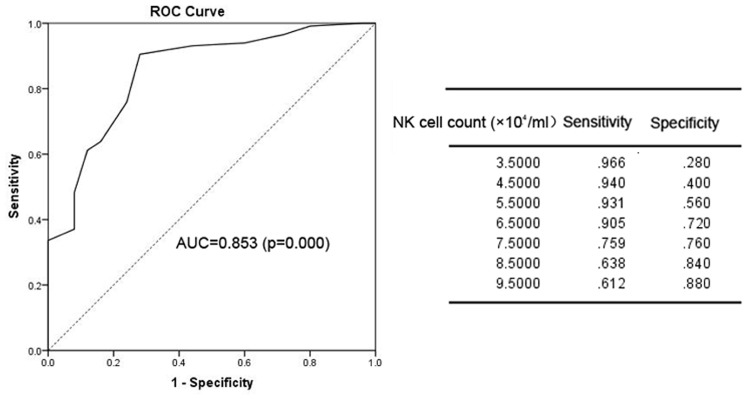
The ROC analysis of the NK cell count. The ROC curve and statistics table for the cut-off value of the NK cell count in the patients’ peripheral blood for the group of patients with proven and probable invasive infection.

We next sought to determine the optimal timing for beginning anti-fungal prophylaxis by lowering the limit of normal value for the risk factors or using other points that could be used for the initiation of prophylaxis. To solve this problem, we used an ROC curve to identify the cut-off point. The area under the ROC curve (AUC) of IgG was 0.778 (95% CI: 0.660–0.895; *p* = 0.000). The optimal infection point was located between an IgG index of 6.935 mg/mL (sensitivity: 87.9%; specificity: 68.0%; Youden index: 0.559) (**Figure 1**). The AUC of the absolute NK cell count was 0.853 (95%CI: 0.770–0.935; *p* = 0.000). The optimal infection point was located between the absolute NK cell count index of 6.5 × 10^4^/mL (sensitivity: 90.5%; specificity: 72.0%; Youden index: 0.625) (**Figure 2**). Based on this cut-off value, the optimal timing for commencing fungal infection prophylaxis might be IgG < 6.935 mg/mL and an absolute NK cell count < 6.5 × 10^4^/mL. Due to the reference value of IgG and for the ease of remembering, we determined the cut-off point of two risk factors as IgG < 7 mg/mL and an NK cell count < 6.5 × 10^4^/mL in the patients’ peripheral blood. The diagnostic sensitivity and specificity for IgG were 86.2 and 68.0%. The positive predictive value (PPV) and negative predictive value (NPV) were 36.73 and 95.8% respectively. The diagnostic sensitivity and specificity for the NK cell count were 90.5 and 72%, and the PPV and NPV were 41.05 and 97.24%, respectively (**Table 4**).

**Table 4:** Sensitivity, specificity, PPV, and NPV of the cut-off point of risk factors and tests in series and parallel.

Variables	Se (%)	Sp (%)	PPV (%)	NPV (%)
IgG < 7 mg/mL	86.2	68	36.73	95.8
NK count < 6.5 × 10^4^/mL	90.5	72	41.05	97.24
Tests in series (IgG < 7 mg/mL and NK count < 6.5 × 10^4^/mL)	48.0	99.1	92.0	89.84
Tests in parallel (IgG < 7 mg/mL or NK count < 6.5 × 10^4^/mL)	92.0	77.6	46.95	97.83

### Tests in Series and Parallel

We have obtained the cut-off points for two IFI risk factors: IgG < 7 mg/mL and NK cell count < 6.5 × 10^4^/mL in the patients’ peripheral blood. We then applied methods to increase the sensitivity and specificity of the diagnostic test. For this purpose, IgG < 7 mg/mL and NK cell count < 6.5 × 10^4^/mL were compared in series (performed only if IgG < 7 mg/mL and NK cell count < 6.5 × 10^4^/mL) and in parallel (performed for all samples and considered IgG < 7 mg/mL or NK cell count < 6.5 × 10^4^/mL). The diagnostic sensitivity and specificity for the tests performed in series were 48.0 and 99.1%, and the PPV and NPV were 92.0 and 89.84%, respectively. For the tests performed in parallel, the sensitivity and specificity were 92.0 and 77.6%, and the PPV and NPV were 46.95 and 97.83%, respectively (**Table 4**).

## Discussion

An IFI causes morbidity and mortality among patients with hematological malignancies who receive cytotoxic chemotherapy or an HSCT. Overall, the antifungal targeted therapies resulted in successful outcomes in 60% of the patients. The IFI-attributable mortality rate was higher in HSCT patients than in those receiving chemotherapy alone; however, the difference was not statistically significant (Neofytos et al., 2009). Although the incidence of IFI is increasing among immunocompromised patients, the most important factors influencing the incidence of IFI remains unknown. In immunocompromised patients, only the patients who suffered from agranulocytosis following chemotherapy or from grades III to IV acute graft-versus-host diseases were associated with a definite indication to receive prophylaxis for IFI. Moreover, other immunocompromised patients did not have the necessary criteria or indication to receive prophylaxis. In our study, since our medical center specializes in autologous HSCT, we made several changes to improve the survival of autologous HSCT patients. For example, lymphoma patients were administered rituximab to eliminate any possible residual tumor cells, which showed good efficacy in preventing tumor recurrence. Due to the clearance of B cells in the graft, several patients experienced long-term humoral immune dysfunction. We also administered fludarabine and cladribine to clear the resting tumor cells, which resulted in patients with an impairment in both humoral and cellular immunity (Rousso et al., 2015). Thus, the incidence of IFI was extremely high at our center, even in auto-HSCT patients. At our center, since allogeneic HSCT was usually sibling-matched allo-HSCT, the occurrence of severe acute GVHD and chronic GVHD was not high. Moreover, the incidence of IFI in auto-HSCT and allo-HSCT were similar in our study.

Prophylaxis of IFI for these patients was necessary; however, the timing of prophylaxis remains undetermined. Since unnecessary prophylaxis may induce drug resistance and cost money, we analyzed patients from two medical centers, compared IFI and non-IFI patients following stem cell transplantation and found that the IgG levels and NK cell counts in the peripheral blood of patients exhibiting a close relationship with IFI. An optimal prophylaxis time point of IgG level < 7 mg/mL and NK cell count < 6.5 × 10^4^/mL was determined by an ROC curve analysis. Tests performed in parallel (IgG level < 7 mg/mL or NK cell count < 6.5 × 10^4^/mL) were helpful for increasing the sensitivity and specification of the diagnostic test. One potential direction of future work may be to monitor the immune function of patients undergoing antifungal prophylaxis treatment. We could choose a time point of IgG level < 7 mg/mL or NK cell count < 6.5 × 104/mL in the patients’ peripheral blood as the starting time for anti-fungal prophylaxis. Despite the fact that NK cell function is important for the prevention of IFIs (Stuehler et al., 2015; Psoch et al., 2017), since this was a retrospective study, many patients did not undergo measurements of NK cell function during the follow-up period. Further studies involving larger, prospective clinical trials are currently underway, and we will measure NK cell function and IFI prophylaxis in such future trials to verify the relationship between IgG levels and NK cells in the context of fungal infections.

Due to the sample size of the study, the 95%CI of IgG and NK cell count was wide. We also used a conditional logistic regression to increase the statistic efficiency and obtained the same conclusion. Further conclusions should be verified in larger and prospective trials. Such findings can be used to establish prophylactic treatment for fungal infections and the regimen can be adjusted in accordance with the patients’ immune function, which is not restricted to only patients receiving a stem cell transplantation. Other patients (e.g., those taking long-term immunosuppressive drugs and glucocorticoid administration) also can commence or stop IFI prophylaxis by monitoring immune function.

## Author Contributions

JM collected patients’ data, analyzed data and wrote the manuscript; YH and MW collected patients’ data; and XW and YX conducted this study.

## Conflict of Interest Statement

The authors declare that the research was conducted in the absence of any commercial or financial relationships that could be construed as a potential conflict of interest.

## References

[B1] Camargo J. F. Bhimji A. Kumar D. Kaul R. Pavan R. Schuh A. (2015). Impaired T cell responsiveness to interleukin-6 in hematological patients with invasive aspergillosis. *PLoS One* 10:e0123171. 10.1371/journal.pone.0123171 25835547 PMC4383538

[B2] Chinese Invasive Fungal Infection Working Group (2017). The Chinese guidelines for the diagnosis and treatment of invasive fungal disease in patients with hematological disorders and cancers (the fifth revision). *Zhonghua Nei Ke Za Zhi* 56 453–459. 10.3760/cma.j.issn.0578-1426.2017.06.015 28592049

[B3] De Pauw B. Walsh T. J. Donnelly J. P. Stevens D. A. Edwards J. E. Calandra T. (2008). Revised definitions of invasive fungal disease from the European organization for research and treatment of cancer /invasive fungal infections cooperative group and the national institute of allergy and infectious diseases mycoses study group (EORTC/MSG) consensus group. *Clin. Infect. Dis.* 46 1813–1821. 10.1086/588660 18462102 PMC2671227

[B4] Fleming S. Yannakou C. K. Haeusler G. M. Clark J. Grigg A. Heath C. H. (2014). Consensus guidelines for antifungal prophylaxis in haematological malignancy and haemopoietic stem cell transplantation. *Intern. Med. J.* 44 1283–1297. 10.1111/imj.12595 25482741

[B5] Gavalda J. Len O. San Juan R. Aguado J. M. Fortun J. Lumbreras C. (2005). Risk factors for invasive aspergillosis in solid-organ transplant recipients: a case-control study. *Clin. Infect. Dis.* 41 52–59. 10.1086/430602 15937763

[B6] Jantunen E. Nihtinen A. Volin L. Juvonen E. Parkkali T. Ruutu T. (2004). Candidaemia in allogeneic stem cell transplant recipients: low risk without fluconazole prophylaxis. *Bone Marrow Transplant.* 34 891–895. 10.1038/sj.bmt.1704662 15517009

[B7] Kontoyiannis D. P. Marr K. A. Park B. J. Alexander B. D. Anaissie E. J. Walsh T. J. (2010). Prospective surveillance for invasive fungal infections in hematopoietic stem cell transplant recipients, 2001–2006: overview of the Transplant-Associated Infection Surveillance Network (TRANSNET) Database. *Clin. Infect. Dis.* 50 1091–1100. 10.1086/651263 20218877

[B8] Kriengkauykiat J. Ito J. I. Dadwal S. S. (2011). Epidemiology and treatment approaches in management of invasive fungal infections. *Clin. Epidemiol.* 3 175–191. 10.2147/CLEP.S12502 21750627 PMC3130903

[B9] Kurosawa M. Yonezumi M. Hashino S. Tanaka J. Nishio M. Kaneda M. (2012). Epidemiology and treatment outcome of invasive fungal infections in patients with hematological malignancies. *Int. J. Hematol.* 96 748–757. 10.1007/s12185-012-1210-y 23111539

[B10] Lehrnbecher T. Schmidt S. Tramsen L. Klingebiel T. (2013). Immunotherapy of invasive fungal infection in hematopoietic stem cell transplant recipients. *Front. Oncol.* 3:17. 10.3389/fonc.2013.00017 23404543 PMC3566394

[B11] Lin S. J. Schranz J. Teutsch S. M. (2001). Aspergillosis case-fatality rate: systematic review of the literature. *Clin. Infect. Dis.* 32 358–366. 10.1086/318483 11170942

[B12] Martino R. Subirá M. Rovira M. Solano C. Vázquez L. Sanz G. F. (2002). Invasive fungal infections after allogeneic peripheral blood stem cell transplantation: incidence and risk factors in 395 patients. *Br. J. Haematol.* 116 475–482. 10.1046/j.1365-2141.2002.03259.x 11841455

[B13] Martín-Peña A. Aguilar-Guisado M. Cisneros J. M. (2014). Does the current treatment of invasive fungal infection need to be reviewed? *Enferm. Infecc. Microbiol. Clin.* 32 523–528. 10.1016/j.eimc.2013.02.008 23587702

[B14] Mellinghoff S. C. Panse J. Alakel N. Behre G. Buchheidt D. Christopeit M. (2018). Primary prophylaxis of invasive fungal infections in patients with haematological malignancies: 2017 update of the recommendations of the infectious diseases working party (AGIHO) of the German society for haematology and medical oncology (DGHO). *Ann. Hematol.* 97 197–207. 10.1007/s00277-017-3196-2 29218389 PMC5754425

[B15] Neofytos D. Horn D. Anaissie E. Steinbach W. Olyaei A. Fishman J. (2009). Epidemiology and outcome of invasive fungal infection in adult hematopoietic stem cell transplant recipients: analysis of multicenter prospective antifungal therapy (PATH) alliance registry. *Clin. Infect. Dis.* 48 265–273. 10.1086/595846 19115967

[B16] Oliveira-Coelho A. Rodrigues F. Campos A. Jr. Lacerda J. F. Carvalho A. Cunha C. (2015). Paving the way for predictive diagnostics and personalized treatment of invasive aspergillosis. *Front. Microbiol.* 6:411. 10.3389/fmicb.2015.00411 25999936 PMC4419722

[B17] Pagano L. Caira M. Candoni A. Offidani M. Martino B. Specchia G. (2010). Invasive aspergillosis in patients with acute myeloid leukemia: a SEIFEM-2008 registry study. *Haematologica* 95 644–650. 10.3324/haematol.2009.012054 19850903 PMC2857195

[B18] Psoch W. Steger M. Wilflingseder D. Lass-Flörl C. (2017). Promising immunotherapy against fungal diseases. *Expert Opin. Biol. Ther.* 17 861–870. 10.1080/14712598.2017.1322576 28429626

[B19] Rousso S. Z. Shamriz O. Zilkha A. Braun J. Averbuch D. Or R. (2015). Hematopoietic stem cell transplantations for primary immune deficiencies: 3 decades of experience from a tertiary medical center. *J. Pediatr. Hematol. Oncol.* 37 e295–e300. 10.1097/MPH.0000000000000352 25985240

[B20] Stuehler C. Kuenzli E. Jaeger V. K. Baettig V. Ferracin F. Rajacic Z. (2015). Immune reconstitution after allogeneic hematopoietic stem cell transplantation and association with occurrence and outcome of invasive aspergillosis. *J. Infect. Dis.* 212 959–967. 10.1093/infdis/jiv143 25748323

